# Influence of the H1N1 influenza pandemic on the humoral immune response to seasonal flu vaccines

**DOI:** 10.1371/journal.pone.0258453

**Published:** 2021-10-22

**Authors:** Hyesun Jang, Ted M. Ross

**Affiliations:** 1 Center for Vaccines and Immunology, University of Georgia, Athens, Georgia, United States of America; 2 Department of Infectious Diseases, University of Georgia, Athens, Georgia, United States of America; University of South Dakota, UNITED STATES

## Abstract

In this study, we hypothesized that the humoral response to trivalent seasonal influenza virus vaccines was influenced by rapid antigenic switching of H1 HA. We tested archived sera and peripheral blood mononuclear cells (PBMC) collected at prior to vaccination at day 0, as well as days 30 and 90 after vaccination during the 2009/2010 and 2010/2011 influenza virus seasons. During the 2009/2010 season, vaccination successfully induced antibodies with hemagglutinin inhibition (HAI) activity against both H1N1 and H3N2 vaccine components. For the 2010/2011 season, the A/California/04/2009 (CA/09) H1N1 elicited seroconversion (HAI titer = 1:40) and novel memory B cell (B_mem_) responses from most individuals. However, the H3N2 influenza virus component of the vaccine, A/Perth/16/2009 (Perth/09), back-boosted and elicited antibodies with HAI activity and B_mem_ response to historical H3N2 influenza virus strains. Following stratification of the pre-existing antibody with HAI against the CA/09 H1N1, there was a negative correlation with HAI seroconversion to other vaccine strains. Overall, strong immune responses against CA/09 H1N1 influenza virus negatively influenced the induction of novel humoral responses.

## Introduction

A novel H1N1 influenza virus (pH1N1) emerged in North America in March 2009 [1]. This virus rapidly spread rapidly among the immunologically naïve human population and the World Health Organization (WHO) declared a phase 6 pandemic in June 2009 (H1N1pdm09). As the second wave of H1N1 pmd09 overlapped with the 2009/2010 influenza season, a monovalent vaccine was deployed in addition to the trivalent vaccine. Later in February 2010, the WHO published recommendations for 2010/2011 influenza vaccines to include the pH1N1-like virus, A/California/04/09 (CA/09 H1N1), in the trivalent vaccine formulation.

Since the novel pH1N1 influenza virus shared common epitopes with previously circulating H1N1 strains, pre-existing memory B or T cells can be recalled by exposure to the novel H1 HA antigen. As a result, a single 15 μg dose of monovalent pH1N1 influenza virus vaccine elicited antibodies with hemagglutinin inhibition (HAI) activity at a titer (1:40 or higher) for seroconversion in healthy young adults that had not been previously exposed to the pH1N1 influenza virus influenza virus [2].

The effectiveness of seasonal influenza virus vaccines has fluctuated from 19% to 64% since the introduction of the pH1N1 influenza virus into the human population. Of note, the highest vaccine effectiveness recorded in the past ten years coincided with the use of the monovalent pH1N1 influenza virus vaccine deployed during the 2009/2010 influenza virus season, as well as the trivalent seasonal influenza virus vaccine used during the 2010/2011 influenza virus season [3, 4]. Two major epidemiologic findings may account for this phenomenon: 1) the excellent immunogenicity of pH1N1 influenza virus vaccine and 2) pre-existing immunity influencing responsiveness to the monovalent pH1N1 influenza virus vaccine [1, 5]. However, the vaccine effectiveness of the 2009/2010 influenza virus vaccine was not fully evaluated. In this study, we hypothesized that the humoral response induced by the trivalent seasonal influenza virus vaccines used during the 2009/2010 and 2010/2011 influenza virus seasons was influenced by the introduction of novel H1 HA component, derived from pH1N1 virus.

## Materials and methods

### Study approval

The study procedures, informed consent, and data collection documents were reviewed and approved by the IRB of the H-13000 and the University of Messachusetts (MA). The funding source had no role in sample collection, nor decision to submit the manuscript for publication. Subjects were recruited in MA, USA, and enrolled with written, informed consent. Exclusion criteria included documented contraindications to hyper sensitivity to vaccine components, previous life threatening reaction to influenza vaccine, altered immune competence state, neurological disorder; pregnancy; acute febrile illness; history of anemia or bleeding disorders; and donation of 60 cc or higher blood within the past 30 days.

### Study participants

**Fig 1:**
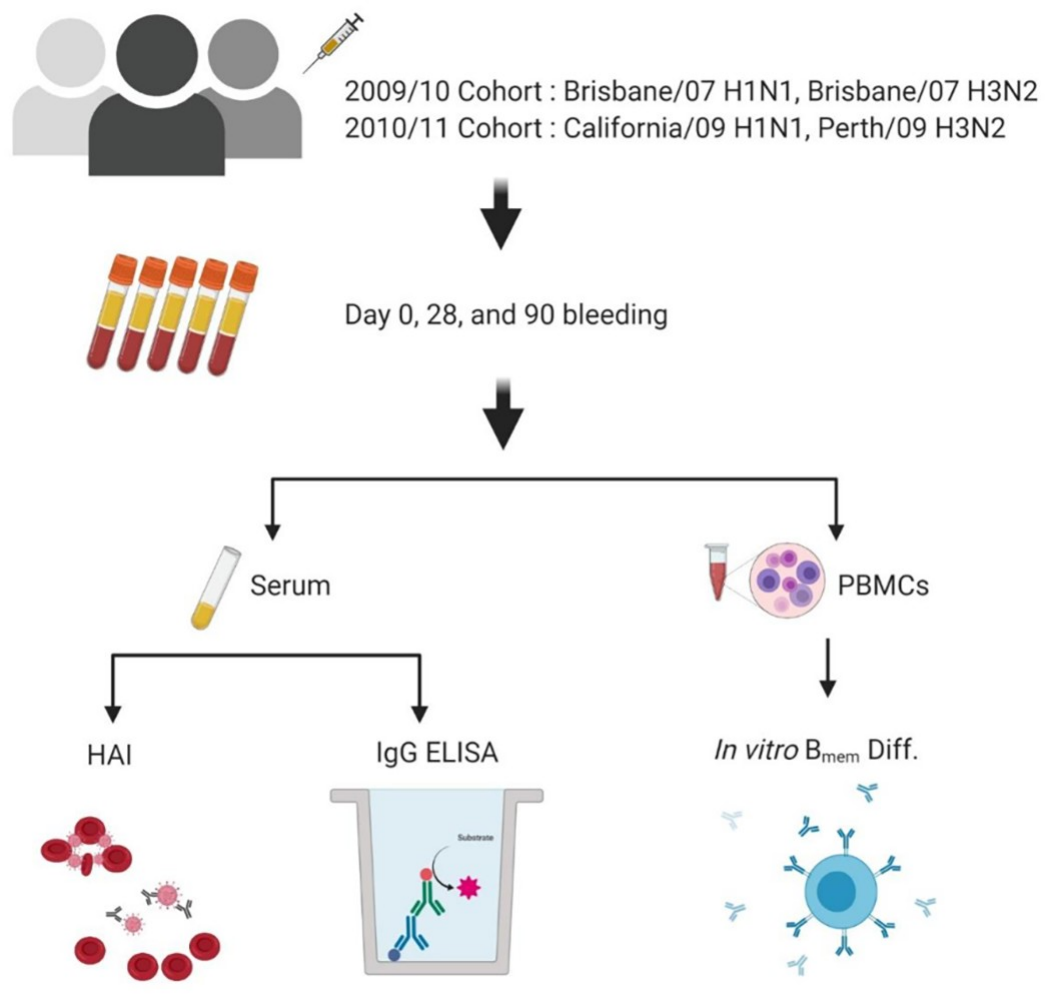
Experimental design. Healthy volunteers were vaccinated with the standard dose (15 μg/antigen) split-virion (IIV) version of licensed Fluzone (Sanofi Pasteur). Serum and PBMCs samples were collected prior to (Day 0), 21–28 days (Day 28), and ~3 months (day 90) following vaccination. Hemagglutination inhibition activity and total HA-specific IgG were measured in serum samples collected at day 30 and 90. The PBMC samples collected at day 0, 30 and 90 were differentiated *in vitro*, and conditioned supernatants were tested for reactivity against the 4 vaccine components to quantify the memory-derived antibody response. Figure was created with *BioRender*.*com*.

During the 2009/2010 and 2010/2011 northern hemisphere influenza seasons, 30 eligible participants were consented and enrolled in the study. Demographic information was only available for the 2009/2010 chorot (Table 1). Monovalent pH1N1 vaccine was give in November and December 2009 to study participants, which was earlier than sample collection for this study (Table 1). All participants were 18–45 years of age. Blood (70–90 mL) was collected from each subject at the time of vaccination (D0) and collected again 21–30 days (D28) and 83–90 days (D90) after vaccination. Each participant was vaccinated with a 15ug standard dose of trivalent split-inactivated influenza vaccine (FluZone^®^, Sanofi Pasteur, Swiftwater, PA, USA). Blood samples were processed for sera and PBMC. For PBMC isolation, blood was collected in Vacutainer CPT tubes (BD Biosciences) at D0, D7, and D21. These samples were processed within 6 hours of collection and stored at liquid nitrogen (vapor phase) for future analysis. Serum was collected in Vacutainer SST tubes (BD Biosciences) and processed within 24–48 hours, stored at 4°C until separated, and aliquoted for long-term storage at –20°C. Overall workflow for each sample and time point is summarized in Fig 1.

**Table 1:** Demographic information of the 2009/2010 cohort.

Subject	Date of Birth	Gender	monovlaent pH1N1 vaccine	Natrual infection
1	9/18/1968	F	No	No
2	5/12/1971	F	No	No
3	8/18/1965	F	No	No
4	12/23/1968	F	No	No
5	9/30/1965	F	No	No
6	5/23/1970	F	No	No
7	4/20/1982	F	No	No
8	9/26/1976	M	No	No
9	6/22/1976	F	No	No
10	1/21/1964	F	Received in Oct 2009	No
11	8/24/1985	M	Received in Nov 2009	No
12	6/15/1985	F	Received in Dec 2009	No
13	11/17/1964	F	Received in Oct 2009	Infected in Nov 2009
14	12/3/1976	F	No	No
15	6/22/1983	M	No	No
16	9/10/1969	F	No	No
17	12/5/1973	M	No	No
18	12/5/1973	M	No	No
19	1/23/1969	F	Received in Nov 2009	No
20	4/23/1966	F	Received in Nov 2009	No
21	5/2/1985	F	No	No
22	6/2/1989	M	Received but no date	No
23	12/28/1970	F	Received in Nov 2009	No
24	9/24/1984	M	No	No
25	11/30/1981	M	Received in Nov 2009	No
26	5/15/1986	M	Received in Nov 2009	No
27	5/30/1977	F	No	No
28	11/2/1983	F	Received in Nov 2009	No
29	3/19/1968	M	No	No
30	11/13/1966	M	Received in Nov 2009	No

### Hemagglutination inhibition assay (HAI assay)

HAI assay was conducted in accordance with the protocols from the WHO laboratory influenza surveillance manual. As a testing antigen, influenza viruses were obtained through the Influenza Reagents Resource (IRR), BEI Resources, or the Centers for Disease Control and Prevention (CDC) and propagated in 10-day-old embryonated, specific pathogen-free (SPF) chicken eggs. Tested serum was treated with receptor-destroying enzyme (RDE) (Denka Seiken Co.) prior to being tested in accordance with manufacture’s recommendation. RDE-treated sera was serially diluted in PBS 2-fold across v-bottom microtiter plates (50 μL/well) and an equal volume of each influenza virus (8 HAu/50 μL l) was added. The plates were covered and incubated at RT for 20 minutes, and then 0.8% turkey erythrocytes (Lampire Biologicals) were added to the virus-serum mixture and incubated for 30 minute at room temperature. The HAI titer was determined by the reciprocal dilution which inhibited agglutination of TRBCs. People were considered seronegative with a titer less than 1:40.

### ELISA

HA binding IgG level was measured by ELISA as previously described [6]. Briefly, a high-affinity, 96-well flat bottom enzyme-linked immunosorbent assay (ELISA) plate was coated with 100 ng of a recombinant H3 antigens generated in house as previously described [6] in ELISA carbonate buffer (50 mM carbonate buffer, pH 9.5). The coated plates was incubated overnight at 4°C and washed in PBS with 0.05% Tween 20 (PBST). Nonspecific epitopes were blocked with 1% bovine serum albumin (BSA) in PBST solution for 1 h at room temperature (RT) and serially diluted serum samples were added (100 μL /well). After two hour incubation at 37°C, plates were washed and probed with goat anti-human IgG horseradish peroxidase-conjugated secondary antibody at a 1:3000 dilution and incubated for 2 h at 37°C. Plates were washed 7 times with the wash buffer prior to development with 100 μL of 0.1% 2,2’-azino-bis(3-ethylbenzothiaozoline-6 –sulphonic acid; ABTS) solution with 0.05% H_2_O_2_ for 40 min at 37°C. The reaction was terminated with 1% (w/v) sodium dodecyl sulfate (SDS). Colorimetric absorbance at 414 nm was measured using a PowerWaveXS (Biotek, Winooski, VT, USA) plate reader. Background was subtracted from negative wells. Linear regression standard curve analysis was performed using the known concentrations of recombinant standard antigen to estimate the HA content in VLP lots.

### *In vitro* differentiation of B cells

PBMCs were plated on 12-well cell culture plate (2 × 10^6^ viable cells/mL) with complete media containing RPMI 1640 medium (MilliporeSigma) with 10% FBS (Atlanta Biologicals), 23.8 mM sodium bicarbonate (Thermo Fisher Scientific), 7.5 mM HEPES (Amresco), 170 μM Penicillin G (Tokyo Chemical Industry), 137 μM Streptomycin (MilliporeSigma), 50 μM 2-mercaptoethanol (MilliporeSigma), 1 mM sodium pyruvate (Thermo Fisher Scientific), essential amino acid solution (Thermo Fisher Scientific), nonessential amino acid solution (Thermo Fisher Scientific), 500 ng/mL R848 (Invivogen), and 5 ng/mL rIL-2 (R&D Systems) for 7–9 days at 37°C in 5% CO2 [6]. Conditioned medium supernatants were harvested and evaluated for total and rHA-specific IgG abundance by ELISA starting at a 1:5 dilution.

### Statistics

Values were considered significant for P<0.05. Statistical significance for HAI titer, rHA-binding IgGs, and B_mem_ derived IgGs was calculated using 2-way ANOVA Tuckey’s multiple comparison test for pairwise comparison between day 0 and day 28. Correlation analysis on B_mem_ derived IgGs was determined by Pearson’s r correlation assay.

All statistical analyses were performed using GraphPad Prism V.8.01 software.

## Results

### People vaccinated with an influenza virus vaccine in 2009/2010 and 2010/2011 had antibodies with hemagglutination inhibition (HAI) activity against the homologous H1N1 virus both influenza seasons

**Fig 2:**
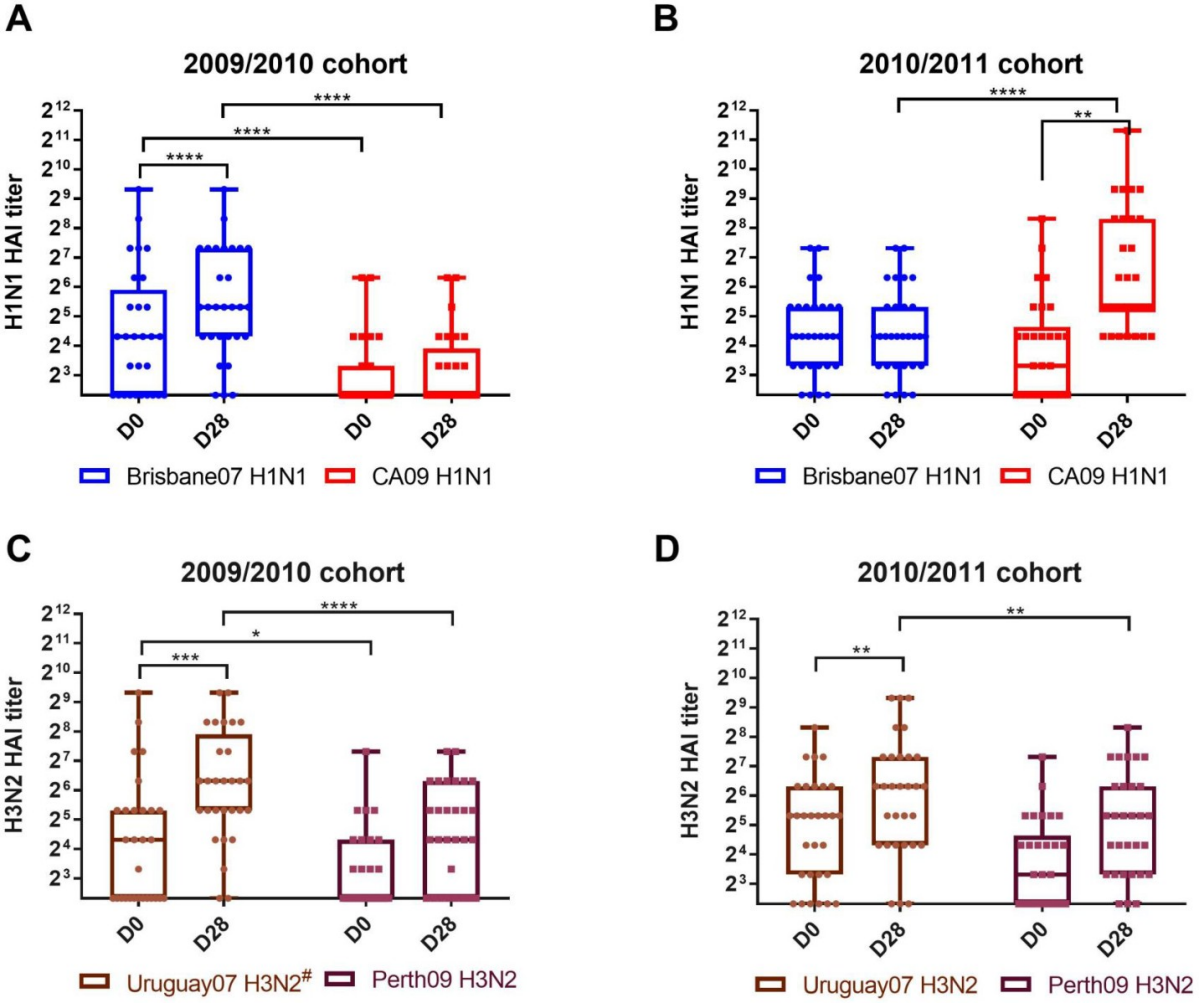
Serum HAI antibody response to vaccinations. (A&B) HAI response to H1N1 or H3N2 components during 2009/2010 influenza seasons, respectively. (C&D) HAI response to H1N1 or H3N2 components during 2010/201 influenza seasons, respectively. Serum samples collected at day 0 and 28 were tested for the HAI antibody response specific four vaccine viruses. Individual titer was plotted. Box plot indicates 10 to 90 percentile and middle line represents median value. The whiskers go down to the smallest value and up to the largest. ***<0.001, ****<0.0001.

During the 2009/2010 season, 18 out of the 29 participants had pre-existing antibodies with HAI activity against Bris/07 H1N1 vaccine component (median HAI titer = 20) (Fig 2A). In contrast, only eight participants had pre-existing antibodies with HAI activity (median HAI titer = 0) against the CA/09 pH1N1 virus (Fig 2A). After vaccination, most participants (26/29) seroconverted to the Bris/07 H1N1 influenza virus with an increase in the median HAI titer (1:40) by day 30 post-vaccination (Fig 2A). There was no increase in HAI titer against CA/09 influenza virus following vaccination which was significantly lower than the HAI titer against Bris/07 (p <0.0001).

Participants vaccinated during the 2010/2011 influenza virus season had a higher pre-existing HAI titer against the CA/09 H1N1 than during the prior season (Fig 2B). Approximately 50% of the participants (17/30) had pre-existing antibodies with HAI activity against the CA/09 influenza virus (Fig 2B). The median HAI titer against the Bris/07 H1N1 influenza virus at day 0 was 1:20, which was the same as the previous influenza season (Fig 2A and 2B, respectively). After vaccination, there was a significant four-fold increase (p <0.0001) in the median HAI titer against the CA/09 influenza virus (Fig 2B). However, the HAI titers against Bris/07 was statistically similar to the titers of day 0 (Fig 2B).

### The serum HAI response to the H3N2 influenza virus components in both influenza seasons

During the 2009/2010 influenza season, 16 out of the 29 participants had pre-existing antibodies with HAI activity (median HAI titer = 20) against Uru/07 (Fig 2C). Amongst the 16 positive serum samples, 12 samples also had HAI activity against the Perth/09 virus. After vaccination, the HAI titer increased in most participants (25/29) against the H3N2 vaccine component (Uru/07). The median HAI titer was also increased from 1:20 to 1:80 (Fig 2C). While the HAI titers against Perth/09 increased each season following vaccination, the HAI titers were significantly lower than those against Uru/07 (p = 0.0002) (Fig 2C). During the 2010/2011 season, there was a slight increase in the pre-existing HAI titers against Perth/09 H3N2 influenza virus that was similar to the pre-existing HAI titer against Uru/07 (Fig 2D). There was not a statistical increase in the Perth/09 HAI titers following vaccination, even though the vaccine included the Perth/09 vaccine, whereas there was a significant (p = 0.0032) increase in HAI titers against Uru/07 (Fig 2D). Approximately 33% of the 2010/2011 participants (10/30) did not have any increase in HAI titers against Perth/09. Among the 20 individuals that had an increase in Perth/09 H3N2 HAI titers, 14 individuals also had an increase in HAI titers against Uru/07. Following vaccination, HAI titers against the Uru/07 H3N2 virus were significantly higher than the HAI titers against the Perth/09 H3N2 and in both 2009/2010 (p<0.0001) and 2010/2011 (p<0.0044) seasons (Fig 2C and 2D).

### Pre-existing CA/09 H1N1 HAI titer negatively influenced to all four vaccine strains

**Fig 3:**
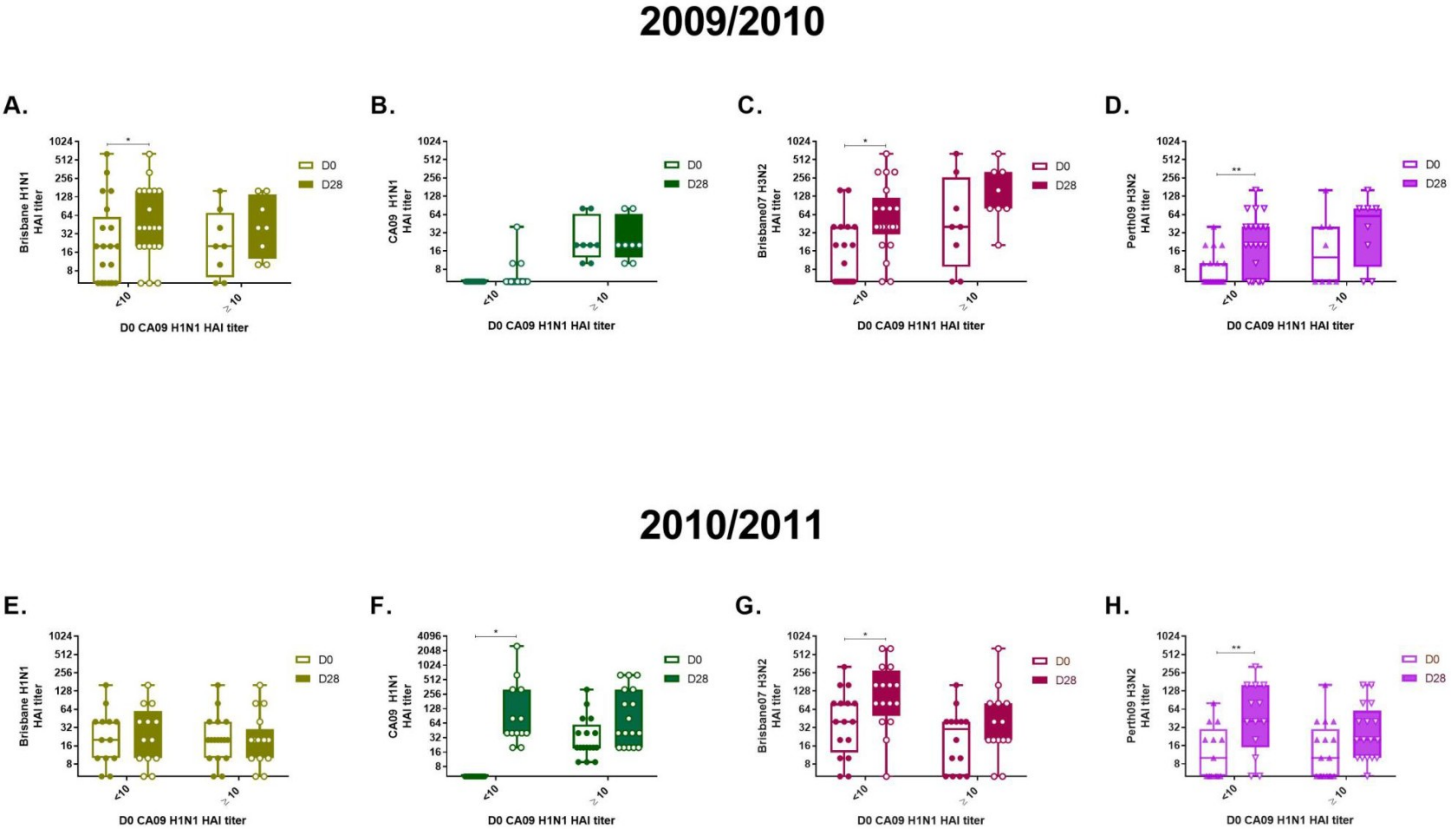
Impact of pre-existing CA09 H1N1 immunity to other subtypes. (A to D) Stratified HAI titer against four vaccine strains following 2009/2010 vaccination. (E to H) Stratified HAI titer against four vaccine strains following 2009/2010 vaccination Serum HAI titer against four vaccine strains were stratified by the presence of D0 HAI antibodies against CA09 H1N1 to investigate the influence of CA09 H1N1 specific pre-existing immunity. Lower detection limit of the HAI assay was 1:10. The whiskers go down to the smallest value and up to the largest. *p<0.05, **p<0.01.

Pre-existing serum antibodies with HAI activity against the CA/09 H1N1 influenza virus may indicate that individuals were exposed to the virus by natural infection or monovalent vaccine deployed during between June and December, 2009. To evaluate the impact of prior exposure to the CA/09 H1N1 antigen, participants were stratified by pre-existing serum HAI titer at D0 prior to vaccination. Among subjects who received CA/09 H1N1 virus vaccination during the 2010/2011 influenza season, only 8 out of the 17 subjects with pre-existing CA/09 H1N1 HAI titers seroconverted to the CA/09 H1N1 virus (Fig 3F). In contrast, all subjects without pre-existing HAI activity to the CA09 virus seroconverted (Fig 3F). Also, those subjects without pre-existing CA/09 H1N1 HAI antibody also had a significant increase in serum antibodies with HAI activity against the Perth/09 H3N2 virus (Fig 3H), but not from those with pre-existing HAI activity to CA/09. For each vaccine strain in both seasons, any significant increase in HAI activity was only observed in serum samples collected from people without pre-existing HAI activity against the CA/09 H1N1 influenza virus (Fig 3). Consistent negative correlation with pre-existing HAI antibody was only observed for the CA/09 H1N1. For example, when stratifying by pre-existing Bris/07 H1N1 HAI titer, we observed an opposite trend; the significant increase in HAI response to H3N2 strains and CA/09 H1N1 was observed from individuals with pre-existing Bris/07 H1N1 HAI antibodies (S1A Fig). The effect of pre-existing Uru/07 H3N2 HAI titer was not consistent during tow influenza seasons; for the 2009/2010 influenza season, pre-existing antibody to the Uruguy/07 H3N2 appear to be negatively correlated with seroconversion to other strains (S1B.A–S1B.D Fig), while significant seroconversion to CA/09 H1N1, Bris/07 and Perth/09 H3N2 was only observed from individuals with pre-existing antibodies to the Uruguay/07 H3N2 (S1B.E–S1B.H Fig). Pre-existing Perth/09 H3N2 appear to negatively correlate to seroconversion of 2009/2010, but not to the 2010/2011 cohort (S1C Fig).

### IgG antibody binding titers against the H3 HA proteins increased following vaccination

**Fig 4:**
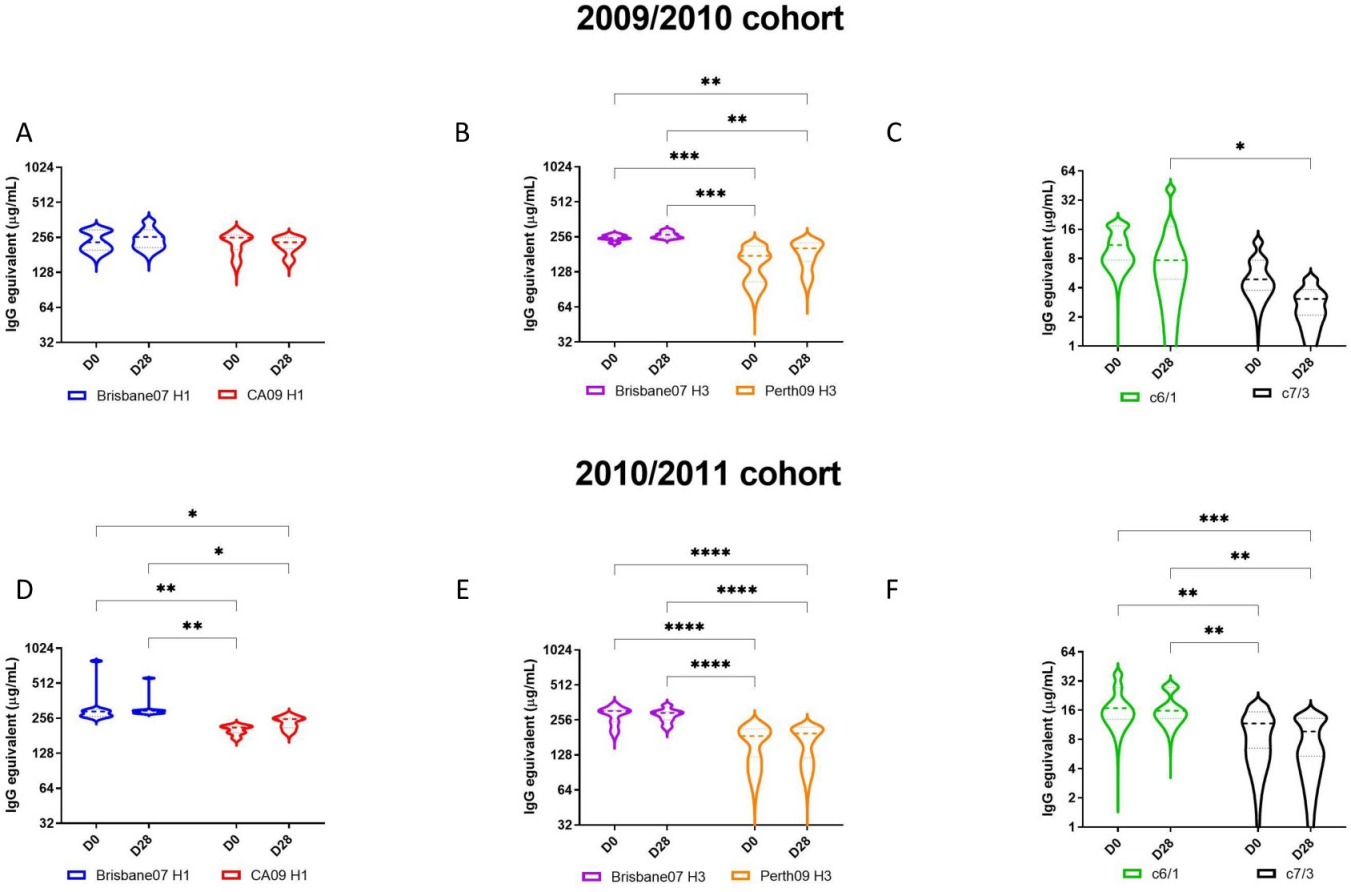
Serum anti-HA binding IgG response. (A, B, D & E) rH1 and rH3 binding IgG titers during two consecutive influenza seasons. Total anti-HA or stem binding IgG response to four vaccine strains was measured by ELISA against four recombinant HA protein of four vaccine strains. (C&F) Stem binding IgG titer during two consecutive influenza seasons. Chimeric HA proteins which retain HA2 stem sequences derived from each strains, but whose HA1 head domain was replaced with those from H6 or H7 HAs (c6/1 and c7/3, respectively). The violin plot illustrated 10 to 90 percentile and middle line represents median value, with emphasis on data distribution. *p<0.05, **p<0.01, ***<0.001, ****<0.0001.

During the 2009/2010 influenza season, the median IgG titer to the Bris/07 H1 rHA and Bris/07 H3 rHA were 232.71 and 250.68 μg/ml prior to vaccination at D0, respectively (Fig 4A and 4B). The medianHA binding IgG titer to the CA/09 H1 rHA and Perth/09 H3 rHA were 255.42 and 408.78 μg/ml, respectively (Fig 4A and 4B). The IgG binding titers prior to vaccination at day 0 against the Bris/07 H3 rHA was significantly higher than to the Perth/09 H3 rHA (p = 0.0009). After vaccination, there was no significant increase in IgG titer for all four strains (Fig 4A–4C). A similar trend was observed during the 2010/2011 season (Fig 4D–4F). There was no significant rise in IgG binding antibodies against both H3 rHA proteins in participants from the 2010/2011 season (Fig 4B and 4E). There was no change in antibody binding titers against the stem portion of the either H1 or H3 HA, but antibodies against the Group 1 stem (c6/1) were in general significantly higher than against the Group 2 stem (c7/3) (Fig 4C and 4F).

### Vaccination induced a significant increase in memory B cell (B_mem_) responses against CA/09

**Fig 5:**
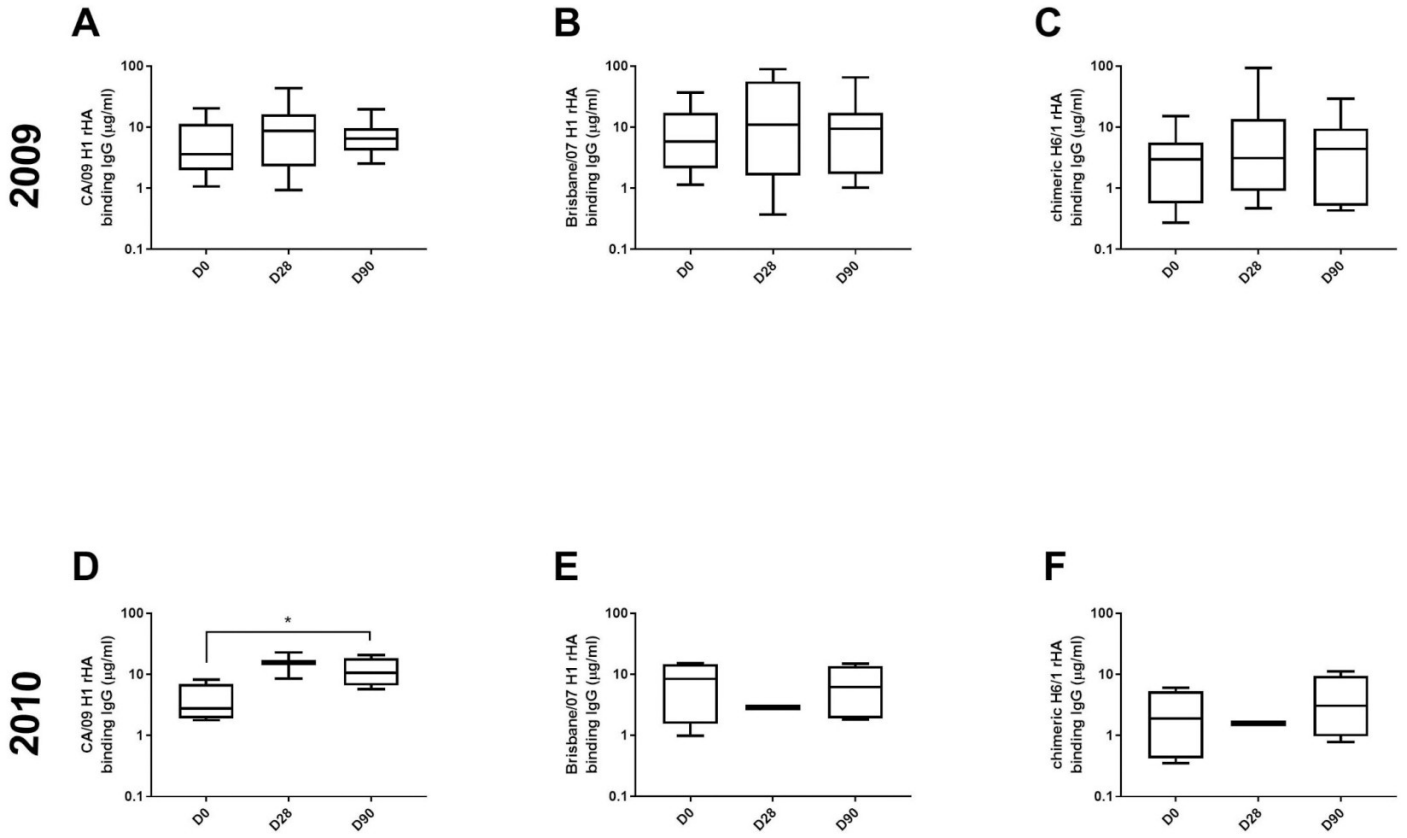
H1 HA specific memory response to vaccinations during 2009/2010 influenza seasons (A to C) and 2010/2011 influenza seasons (D-F). Levels of memory B cell–derived (B_mem_-derived) antibody (IgG) to vaccination was measured by ELISA following in vitro differentiation of PBMCs collected at day 0, 28 and 90 following vaccinations. Box plot indicates 10 to 90 percentile and middle line represents median value. The whiskers go down to the smallest value and up to the largest. *p<0.05.

Long-term protection against the vaccine antigens was measured by the B*mem* response. The HA-specific B_mem_ response was quantified by measuring the IgG antibody titers produced by *ex-vivo* stimulated peripheral blood mononuclear cells (PBMC) collected on days 0, 30, and 90 (Fig 5). Total B_mem_-derived IgG (B_mem_-IgG) from each three time point was controlled to be at the similar level (S2 Fig).

Interestingly, in the 2010/2011 season, the CA/09 H1 HA-specific B_mem_-IgG titer significantly rose (p = 0.0114) from D0 levels by five-fold at day 90 post-vaccination (Fig 5D). There was no significant change in Bris/07 H1 HA-specific B_mem_-IgG titers in the 2010/2011 participants (Fig 5F).

The chimeric cH6/H1 HA B_mem_-derived IgG was also measured to differentiate recall responses targeting the stem portion of the HA protein, which is shared between Bris/07 and CA/09 H1 HAs (Fig 5C and 5F). Overall, the CA/09 and Bris/07 B_mem_-IgG titer against the cH6/H1 HA was lower than H1 HA-specific B_mem_-IgG titer (Fig 5C and 5F). In both cohorts, there was no significant change in cH6/H1 HA-specific B_mem_-IgG response among three different time points. Interestingly, cH6/H1 HA-specific B_mem_-IgG response showed a similar trend with Brisbane07 H1 HA-specific B_mem_-IgG response in the 2010/2011 cohort (Fig 5E & 5F).

### Memory B cell derived IgGs (B_mem_–IgGs) preferentially bound to the Uru/07 H3 rHA and historical H3N2 vaccine strains

**Fig 6:**
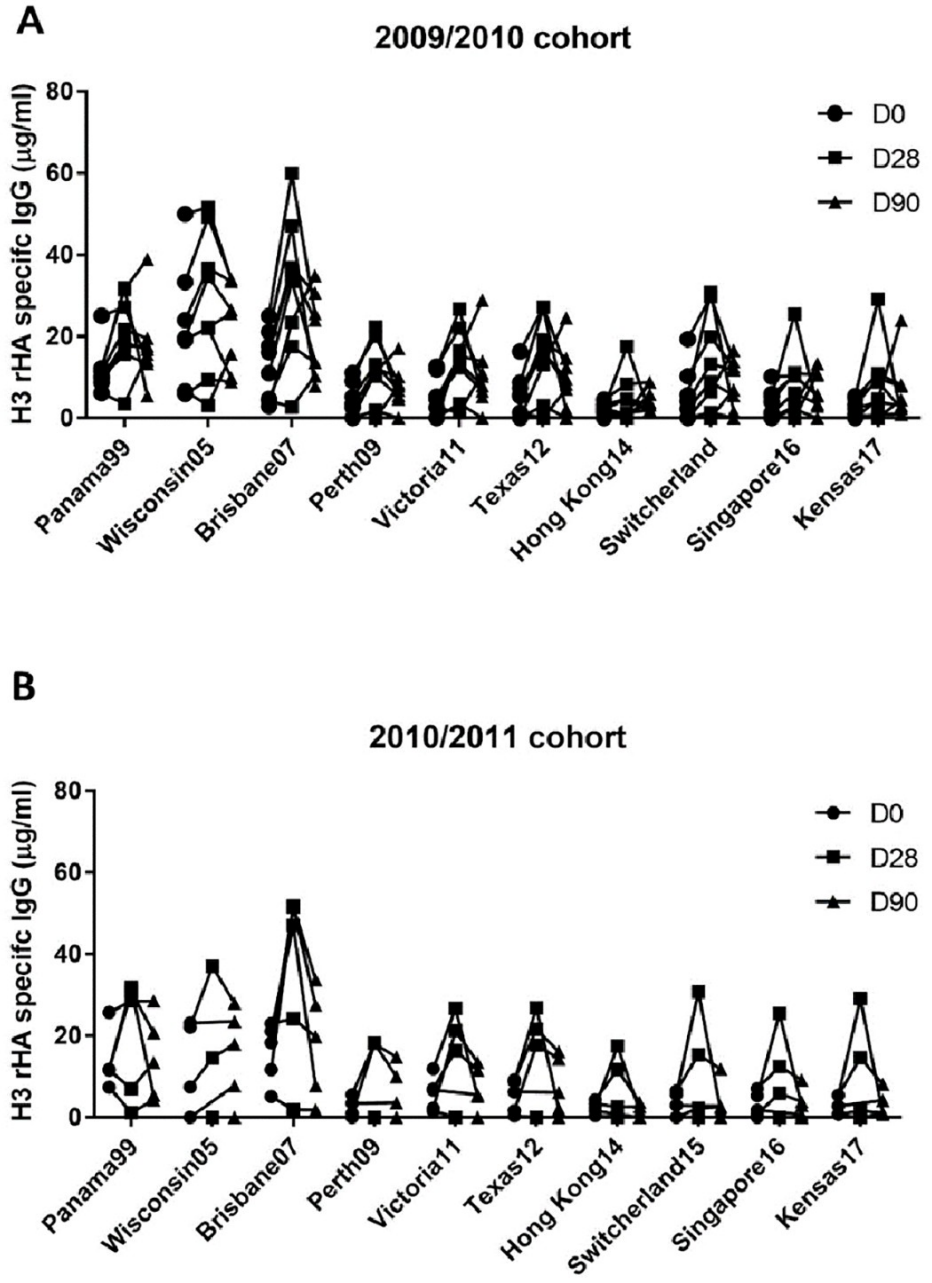
H3 HA specific memory response to vaccinations during 2009/2010 influenza seasons (A) and 2010/2011 influenza seasons (B). Levels of memory B cell–derived (B_mem_-derived) antibody (IgG) to vaccination was measured by ELISA following in vitro differentiation of PBMCs collected at day 0, 28 and 90 following vaccinations. Box plot indicates the smallest to highest B_mem_-derived IgG equivalent.

The Cross-reactive, pre-existing immunity to the H3 HA strains influenced the B_mem_-IgG induced antibodies against historical H3 HA proteins incorporated into the vaccine over the last two decades. In both seasons, people with pre-existing B_mem_-IgG antibodies had higher titer against the three vaccines strains: Pan/99, Wisc/05, and Bris/07, than against other H3 HA proteins circulating later than 2008 (Fig 6). After vaccination, the B_mem_ -IgG reactivity increased against all H3 panel HA proteins (Fig 6). The B_mem_-IgG levels at D0 and D90 were higher against the three HA proteins circulating before 2008. Interestingly, both Bris/07 and Perth/09 H3N2 vaccinations induced the highest B_mem_-IgG responses against the Bris/07 HA at day 30, which is a consistent trend with the serum HAI activity (Fig 3).

## Discussion

The CA/09 H1N1 induced serum antibodies with HAI activity and B_mem_ secreted IgG from people vaccinated during the 2010/2011 season. The monovalent pH1N1 vaccine induced a protective immune response in immunologically naïve populations. It is possible that the participants might have been previously exposed to the monovalent pH1N1 vaccines or natural infection. During 2010/2011 influenza seasons, more people showed pre-existing HAI titer to CA/09 H1N1 and it supports the hypothesis that some participants were exposed to the antigen (Fig 2B). However, this hypothesis does not fully explain the phenomenon that; 1) the magnitude of serum HAI response to the CA/09 virus was higher than the titer in the previous season (Fig 2A and 2B) and 2) that individuals with pre-existing antibodies against the CA09 HA protein were less responsive (Fig 3). The serum antibodies with HAI activity against the CA/09 virus were associated with the cross-reactive CD4+ T cell responses [7]. For monovalent pH1N1 vaccine, the CD4+ T cell response was directly correlated with the serum neutralizing antibody response [7]. A previous *in silico* study identified that the pdmH1N1 HA protein carries more promiscuous epitopes and are cross-reactive to the pre-existing influenza-specific CD4+ T cells than other seasonal flu strains [8, 9].

The HAI antibodies to Perth/09 is known is poorly reactive to the Uru/07 H3N2 or Bris/07 H3N2-like viruses [10]. It is due to critical mutations introduced on antigenic sites of HA head [10]. In consistent with previous findings, our study confirmed limited range of cross reactivity between two H3N2 strains. Particularly in Fig 2D, the change in mean HAI titer between day 0 and 28 shows similar trend between two H3N2 strains, despite at least 4 fold reduction from Uru/07 to Perth/09 H3N2 HAI titers. The Perth/09 H3N2 vaccine component poorly elicited antibodies with HAI activity against the Perth/09 virus, as well as B_mem_ secreted IgG [10]. Instead, the humoral responses were back-boosted to the Uru/07 H3N2 or historical H3N2 virus vaccine strains (Figs 2 & 6). The 2010/2011 influenza virus season had a bias to the Uru/07 H3N2 vaccine strain due to pre-existing immunity and lower antibody titers that were induced against epitopes on novel drifted viruses. In contrast, the novel H1N1pdm influenza virus still dominated the seasonal influenza virus quasispecies and cross-reactive pre-existing immunity recognized epidemic strains. Previously, the antigenic distance hypothesis (ADH) was suggested that a similar phenomenon using sequential vaccination with antigenically related antigens tends to boost pre-existing immunity to common epitopes [11]. These immune responses hampers or interferes with the elicitation of nascent immune responses to the novel drifted epitopes on HA. As a result, vaccine failure will occur if the vaccine strain is not well-matched with epidemic strains circulating in the human population, as observed during the 2014/2015 influenza season [12]. Recommendations for annual updates of vaccine antigen should consider the potential of negative interference in people when sequentially administrated the same vaccine HA antigen(s).

Stratification of the HAI titers indicated that individuals with pre-existing antibodies with HAI activity against the CA/09 H1N1 virus had fewer immune responses against the H1N1 and H3N2 HA antigens in the influenza vaccine (Fig 3). Previous studies reported this pre-existing immunity has a negative influence on baseline HAI titers induced by vaccination [13–15]. For the monovalent pH1N1 vaccine, sequential administration of same vaccine with the same H1 HA antigen negatively impacts the ability on subsequent vaccinations to elicit effective protective immunity, particularly when used in short intervals (<10 days) [13]. A separate study conducted with health care workers and HIV positive participants also reported that a higher baseline HAI titer prior to vaccination was negatively related to seasonal flu vaccine induce HAI seroconversion and elicitation of interferon-gamma (IFN-γ) responses [14]. The impact of baseline antibodies with HAI activity against the CA/09 H1N1 HA is not limited to the H1N1 subtype.

The negative interference by co-administrated vaccines has been widely investigated in pediatric immunizations since these vaccine schedules administer multiple vaccines simultaneously [16, 17]. One of the most well-known phenomena is that a strong immunogen, such as the CRM197, can lower responsiveness to co-administrated vaccines [16]. While the underlying mechanism is still not clear, it is believed that the co-administrated antigens compete for limited resources within lymph nodes [17]. Similarly, immune interference has been hypothesized during trivalent and quadrivalent seasonal influenza virus vaccinations [18]. The potential for immune interference during trivalent influenza virus vaccination was demonstrated by directly comparing to the elicited immune responses to the immune responses elicited by monovalent vaccine [19]. However, there has been no evidence of immune interference among seasonal influenza virus vaccinations in human. Abreu *et al*. cautiously suggested that one vaccine component could dominate others during the 2017/2018 influenza season [6]. The immune imprinting effect was proposed as an underlying mechanism, but the immune interference by components of the quadrivalent influenza virus vaccine could not substantiated. Our study confirmed that the biased humoral response following trivalent seasonal influenza virus vaccination was not an anecdotal finding and supports the immune interference hypothesis. Future studies will investigate underlying mechanism emphasizing role of cross-reactive Bmem cells, timing of exposure as well as aforementioned hypohtheses.

The limitations to this study including the use of archived serum and PBMC samples collected ~10 years ago. Since the serum and PBMCs were processed separately and assigned independent identification numbers, we could not correlate the individual serum antibody titer with the B_mem_ response. Also, since we could verify subject information only for the 2009/2010 cohort, we could not verify compounding factors, as immunization history, age, or health conditions. Particularly, we could not evaluate the influence of monovalent vaccine or H1N1 infection due to the lack of history on 2010/2011 cohort. Third, the current study was conducted on samples collected from one locality in United States and two-consecutive influenza seasons. Follow-up longitudinal studies are needed to clarify the underlying mechanism and provide better insight. Also, our *ex-vivo* analysis of memory B cell response is limited to non-speicific stimulation only by IL-2. Future studies can extend to explore more antigen specific B_mem_ analysis and more optimal B cell stimulating condition.

Overall, the trivalent seasonal influenza virus vaccination during the 2009 pandemic was effective in inducing antibodies with HAI activity to HA antigens in the vaccine. However, dominant immunogenicity of one vaccine component could negatively influence immune responsiveness to other vaccine components. Seasonal influenza virus vaccinations may be expanded after the COVID-19 pandemic, the impact of sudden antigenic changes and vaccine interference needs to be thoroughly investigated. However, these findings should not be misinterpreted to discourage annual vaccinations as numerous reports still emphasize the importance of yearly immunization [20]. To the contrary, the results of this study supports the investigating the possible antigenic hierarchy among vaccine components and possible negative immune interference effects during influenza virus vaccinations.

## Supporting information

S1 Fig HAI titer against four vaccine strains following 2009/2010 and 2010/2011 vaccination stratified by pre-existing immunity to Brisbane07 H1N1 (A), Brisbane07 H3N2 (B), or Perth09 H3N2 (C). Lower detection limit of the HAI assay was 1:10. The whiskers go down to the smallest value and up to the largest.*p<0.05, **p<0.01.
(PPTX)

S2 Fig Total memory B cell–derived (B_mem_-derived) IgG from nonspecifically stimulated PBMCs. Before analyzing HA-specific Bmem response, the total B_mem_-IgG was confirmed to be the same over three PBMC collection time points. It showed that the change of the B_mem_-IgG level was a result of differentiation of the B cells, not the expansion of total B cells.
(PPTX)

## References

[1] Fineberg H. V. Pandemic preparedness and response—lessons from the H1N1 influenza of 2009. *The New England journal of medicine* 370, 1335–1342, doi: 10.1056/NEJMra1208802 (2014). 24693893

[2] Greenberg M. E., et al. Response to a monovalent 2009 influenza A (H1N1) vaccine. *The New England journal of medicine* 361, 2405–2413, doi: 10.1056/NEJMoa0907413 (2009). 19745216

[3] Griffin M. R., et al. Effectiveness of non-adjuvanted pandemic influenza A vaccines for preventing pandemic influenza acute respiratory illness visits in 4 U.S. communities. *PloS one* 6, e23085, doi: 10.1371/journal.pone.0023085 (2011). 21857999 PMC3155536

[4] Treanor J. J., et al. Effectiveness of seasonal influenza vaccines in the United States during a season with circulation of all three vaccine strains. *Clinical infectious diseases*: *an official publication of the Infectious Diseases Society of America* 55, 951–959, doi: 10.1093/cid/cis574 (2012). 22843783 PMC3657521

[5] Li G. M., et al. Pandemic H1N1 influenza vaccine induces a recall response in humans that favors broadly cross-reactive memory B cells. *Proceedings of the National Academy of Sciences of the United States of America* 109, 9047–9052, doi: 10.1073/pnas.1118979109 (2012). 22615367 PMC3384143

[6] Abreu R. B., Kirchenbaum G. A., Clutter E. F., Sautto G. A. & Ross T. M. Preexisting subtype immunodominance shapes memory B cell recall response to influenza vaccination. *JCI insight* 5, doi: 10.1172/jci.insight.132155 (2020). 31794433 PMC7030826

[7] Skibinski D. A. G., et al. Induction of Human T-cell and Cytokine Responses Following Vaccination with a Novel Influenza Vaccine. *Scientific reports* 8, 18007–18007, doi: 10.1038/s41598-018-36703-7 (2018). 30573748 PMC6301966

[8] De Groot A. S., et al. Low immunogenicity predicted for emerging avian-origin H7N9: implication for influenza vaccine design. *Hum Vaccin Immunother* 9, 950–956, doi: 10.4161/hv.24939 (2013). 23807079 PMC3899161

[9] De Groot A. S., et al. Immune camouflage: relevance to vaccines and human immunology. *Human vaccines & immunotherapeutics* 10, 3570–3575, doi: 10.4161/hv.36134 (2014). 25483703 PMC4514035

[10] Yang J. R., et al. A new antigenic variant of human influenza A (H3N2) virus isolated from airport and community surveillance in Taiwan in early 2009. *Virus research* 151, 33–38, doi: 10.1016/j.virusres.2010.03.011 (2010). 20347893

[11] Smith D. J., Forrest S., Ackley D. H. & Perelson A. S. Variable efficacy of repeated annual influenza vaccination. *Proceedings of the National Academy of Sciences of the United States of America* 96, 14001–14006 (1999). doi: 10.1073/pnas.96.24.14001 10570188 PMC24180

[12] Skowronski D. M., et al. Serial Vaccination and the Antigenic Distance Hypothesis: Effects on Influenza Vaccine Effectiveness During A(H3N2) Epidemics in Canada, 2010–2011 to 2014–2015. *The Journal of Infectious Diseases* 215, 1059–1099, doi: 10.1093/infdis/jix074 (2017). 28180277 PMC5853783

[13] Ohfuji S., et al. Key points in evaluating immunogenicity of pandemic influenza vaccines: A lesson from immunogenicity studies of influenza A(H1N1)pdm09 vaccine. *Vaccine* 35, 5303–5308, doi: 10.1016/j.vaccine.2017.07.092 (2017). 28784284

[14] Agrati C., et al. Cellular and humoral immune responses to pandemic influenza vaccine in healthy and in highly active antiretroviral therapy-treated HIV patients. *AIDS Res Hum Retroviruses* 28, 1606–1616, doi: 10.1089/AID.2011.0371 (2012). 22439734 PMC3505053

[15] Thompson M. G., et al. Effects of Repeated Annual Inactivated Influenza Vaccination among Healthcare Personnel on Serum Hemagglutinin Inhibition Antibody Response to A/Perth/16/2009 (H3N2)-like virus during 2010–11. *Vaccine* 34, 981–988, doi: 10.1016/j.vaccine.2015.10.119 (2016). 26813801 PMC5218812

[16] Findlow H. & Borrow R. Interactions of conjugate vaccines and co-administered vaccines. *Human vaccines & immunotherapeutics* 12, 226–230, doi: 10.1080/21645515.2015.1091908 (2016). 26619353 PMC4962715

[17] Siegrist C. A. Blame vaccine interference, not neonatal immunization, for suboptimal responses after neonatal diphtheria, tetanus, and acellular pertussis immunization. *The Journal of pediatrics* 153, 305–307, doi: 10.1016/j.jpeds.2008.04.032 (2008). 18718256

[18] Lee H., Shim E. H. & You S. Immunodominance hierarchy of influenza subtype-specific neutralizing antibody response as a hurdle to effectiveness of polyvalent vaccine. *Hum Vaccin Immunother* 14, 2537–2539, doi: 10.1080/21645515.2018.1482172 (2018). 29852081 PMC6284502

[19] Jang Y. H., et al. Protective efficacy in mice of monovalent and trivalent live attenuated influenza vaccines in the background of cold-adapted A/X-31 and B/Lee/40 donor strains. *Vaccine* 32, 535–543, doi: 10.1016/j.vaccine.2013.12.002 (2014). 24342248

[20] Trieu M.-C., et al. Augmented CD4+ T-cell and humoral responses after repeated annual influenza vaccination with the same vaccine component A/H1N1pdm09 over 5 years. *npj Vaccines* 3, 37, doi: 10.1038/s41541-018-0069-1 (2018). 30131880 PMC6092382

