# Supplementary figures and images for: Influence of the H1N1 influenza pandemic on the humoral immune response to seasonal flu vaccines
Source: PLoS One. 2021 Oct 22;16(10):e0258453. doi: 10.1371/journal.pone.0258453 (PMC8535392; doi:10.1371/journal.pone.0258453)

## Slide 1
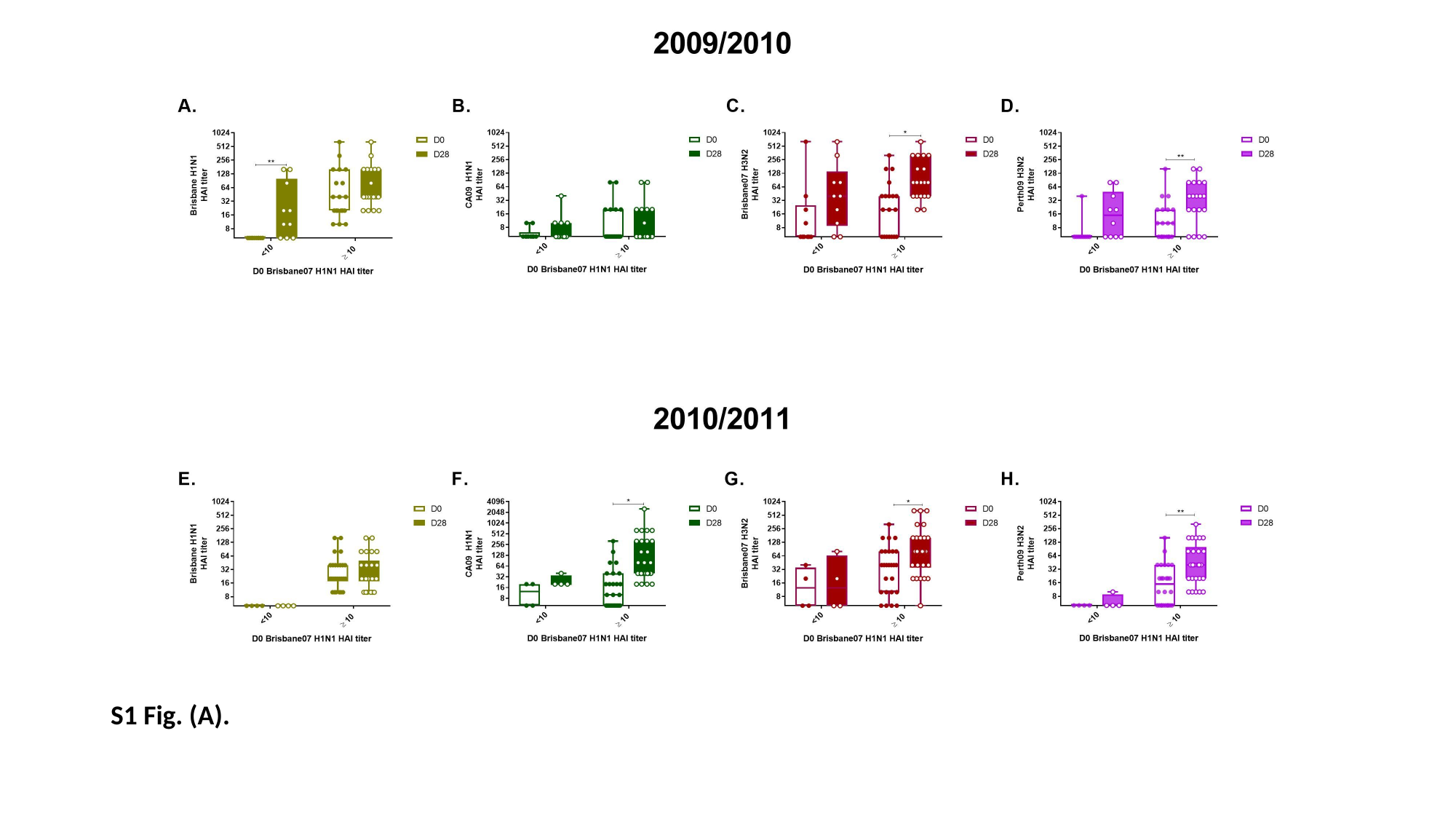

S1 Fig. (A).

## Slide 2
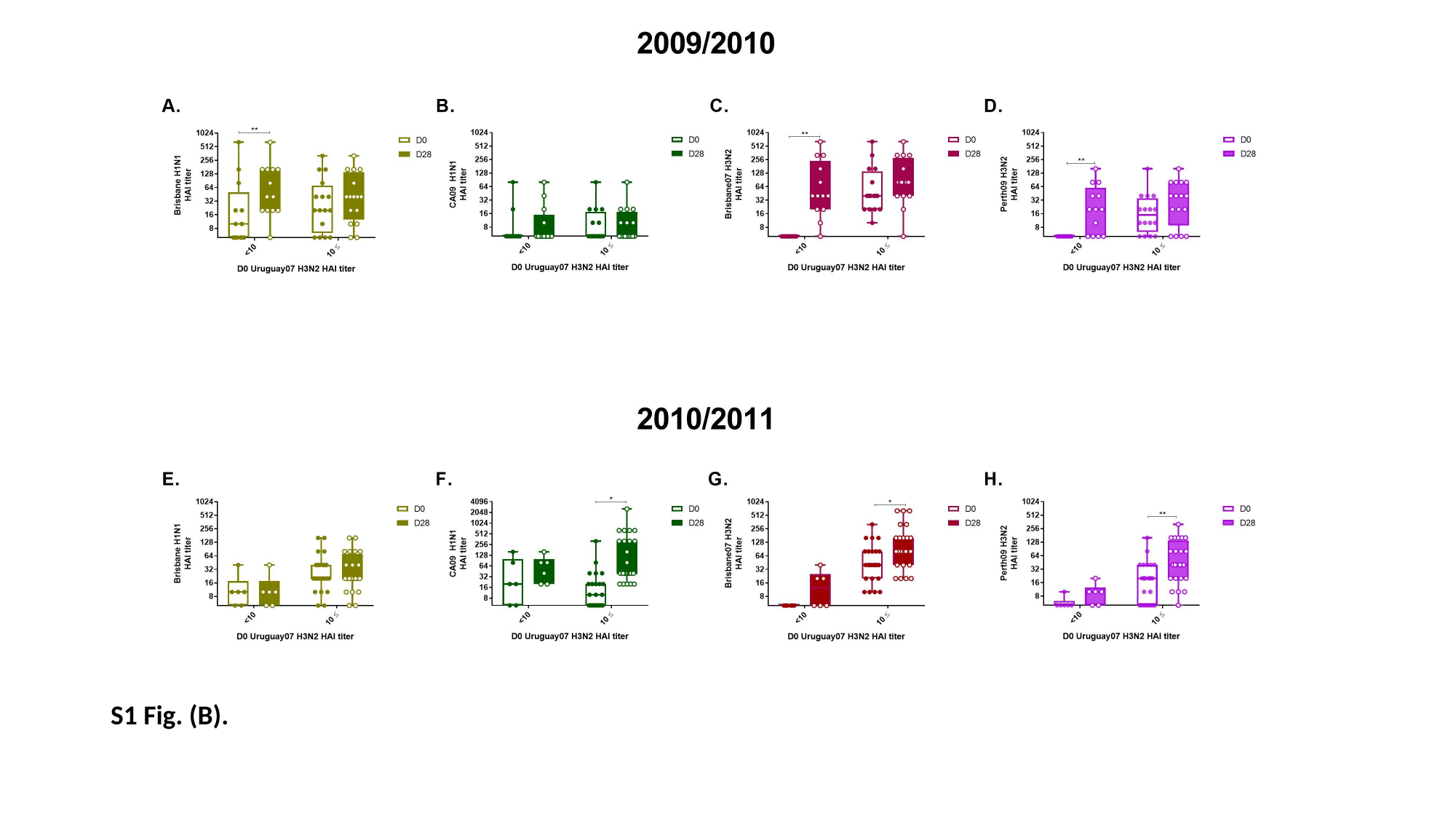

S1 Fig. (B).

## Slide 3
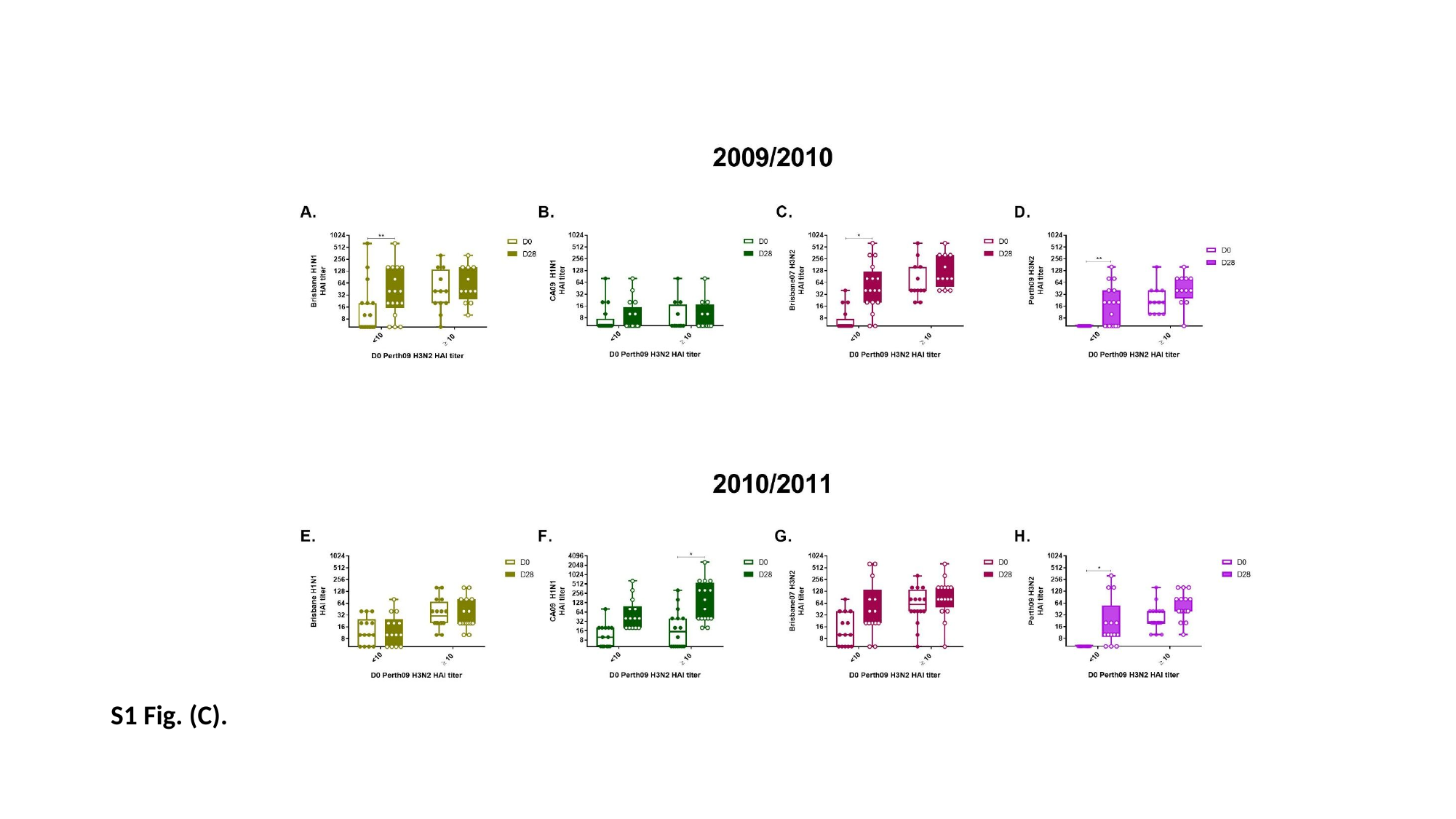

S1 Fig. (C).

Supplement: S1 Fig — Lower detection limit of the HAI assay was 1:10. The whiskers go down to the smallest value and up to the largest.*p<0.05, **p<0.01. (PPTX) [file pone.0258453.s001.pptx]

## Slide 1
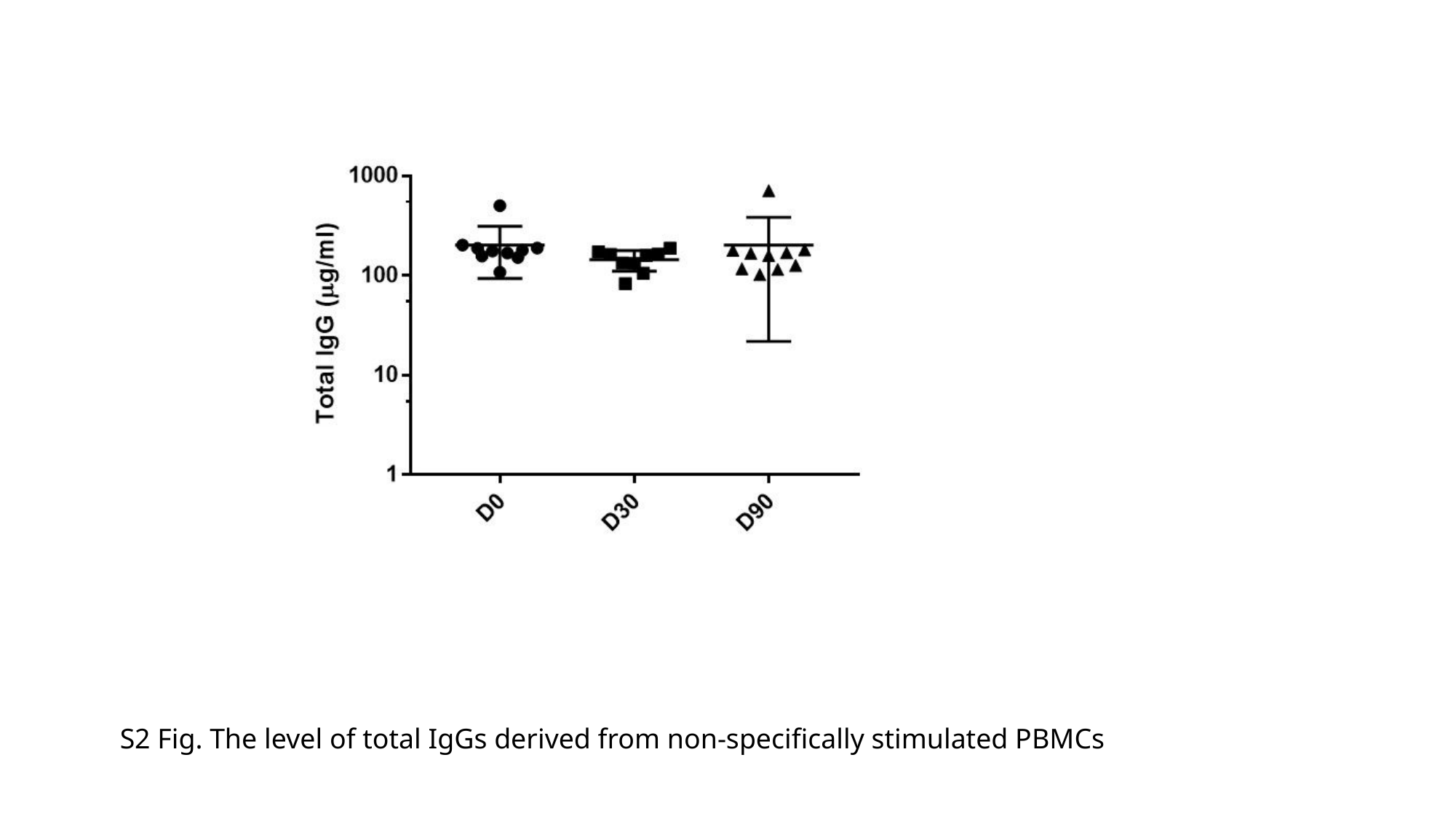

# S2 Fig. The level of total IgGs derived from non-specifically stimulated PBMCs

Supplement: S2 Fig — Before analyzing HA-specific Bmem response, the total Bmem-IgG was confirmed to be the same over three PBMC collection time points. It showed that the change of the Bmem-IgG level was a result of differentiation of the B cells, not the expansion of total B cells. (PPTX) [file pone.0258453.s002.pptx]
